# Care partner evaluation of the behaviors in the Cohen-Mansfield Agitation Inventory

**DOI:** 10.3389/frdem.2024.1328874

**Published:** 2024-03-08

**Authors:** Dorothee Oberdhan, Andrew Palsgrove, Christy Houle, Teya Lovell, A. Alex Levine, Terry Frangiosa, Ginny Biggar, Meryl Comer

**Affiliations:** ^1^Otsuka Pharmaceutical Development and Commercialization, Inc., Global Value and Real-World Evidence, Rockville, MD, United States; ^2^Eli Lilly and Company, Chicago, IL, United States; ^3^Tufts Medical Center, Boston, MA, United States; ^4^Department of Health Law, Policy, and Management, Boston University School of Public Health, Boston, MA, United States; ^5^UsAgainstAlzheimer's, Washington, DC, United States

**Keywords:** patient-centered outcomes, dementia, Alzheimer's disease, agitation, meaningful change

## Abstract

**Introduction:**

Agitation is a common symptom in patients with Alzheimer's dementia. But agitation can be a heterogeneous symptom, encompassing a diverse array of behaviors exhibited by patients. The Cohen-Mansfield Agitation Inventory (CMAI) is a 29-item scale that is used to systematically assess the frequency and severity of agitation in older adults as rated by a primary caregiver. The CMAI was originally designed for use by professional care givers in institutional care settings. Alzheimer's dementia, however, is associated with a significant burden on family members, who provide the majority of care, and other informal care partners.

**Methods:**

Our qualitative study aimed to assess the accuracy and applicability of the CMAI according to the needs and perceptions of non-professional care partners. Specifically, we wanted to determine if the behaviors included in the instrument reflect: (a) the care partner's experience with agitation in Alzheimer's dementia patients, (b) how the behaviors and their frequency are related to the perception of agitation severity, and (c) what changes in agitation behaviors are meaningful to care partners. We interviewed 30 care partners for patients with Alzheimer's dementia in the United States.

**Results:**

The care partners confirmed all behaviors listed in the CMAI as relevant. The behaviors reflect a spectrum of severity, with aggressive behaviors considered more severe than non-aggressive behaviors and physical behaviors generally considered more severe than verbal behaviors. Any reduction or increase in the frequency of a behavior was meaningful to care partners. Generally, a change from physical to verbal behaviors and aggressive to non-aggressive was considered a meaningful improvement while a change from verbal to physical and non-aggressive to aggressive was considered a meaningful worsening.

**Discussion:**

The CMAI appropriately captures relevant behaviors of agitation in Alzheimer's dementia and provides insight into the relative improvement or worsening of agitation symptoms.

## 1 Introduction

Dementia is a neurodegenerative brain disorder with diverse clinical symptoms including cognitive impairment (e.g., memory loss and learning deficits) and non-cognitive symptoms (e.g., behavioral and psychological deficits), leading to functional impairment of activities of daily living. Alzheimer's disease is the most common cause of dementia, with an estimated 60 million people living with the disease and other forms of dementia worldwide; in the US, ~6.5 million Americans aged ≥ 65 years have the condition (Zhao, 2020).

Neuropsychiatric symptoms including agitation are highly prevalent in Alzheimer's dementia (Halpern et al., 2019). These symptoms are experienced across the continuum of disease severity and typically become more serious as the disease progresses (Halpern et al., 2019). Although agitation is a common neuropsychiatric symptom, it was not clearly defined until the International Psychogeriatric Association (IPA) established a provisional consensus definition in 2015 (Cummings et al., 2015). The IPA defined agitation in Alzheimer's dementia as manifestations of excessive motor activity, verbal aggression, or physical aggression that are consistent with emotional distress for the person concerned, cause excess disability, and are not solely attributable to another disorder (Antonsdottir et al., 2015). Agitation is associated with accelerated disease progression, functional decline, decreased quality of life, increased risk of institutionalization, and earlier death (Banerjee, 2006; Scarmeas et al., 2007; Wilcock et al., 2008; Koenig et al., 2015; Lanctôt et al., 2017; Halpern et al., 2019; Rockwood et al., 2019).

The Cohen-Mansfield Agitation Inventory (CMAI) Long Form is a 29-item questionnaire used to assess the frequency of agitation behaviors (Cohen-Mansfield, 1991). The questionnaire is administered to the care partner who provides the majority of the work directly or via interview by a clinician. The frequency of behaviors is based on care partner observation and is rated on a 7-point scale (1 = never; 2 = less than once a week; 3 = once or twice a week; 4 = several times a week; 5 = once or twice a day; 6 = several times a day; 7 = several times an hour). A total CMAI score is obtained by summing all the individual items, giving a range from 29 to 203. Agitation behaviors are heterogeneous and not every patient exhibits all behaviors; therefore, various factor or domain structures have been suggested for the CMAI, depending on population characteristics. For our research, we applied the factors described by Cohen-Mansfield for the community setting as physically non-aggressive, physically aggressive, verbally non-aggressive, and verbally aggressive, which include all 29 items. The CMAI does not include a severity rating for behaviors since a latent severity component is implied by different types of behaviors. For example, aggressive behaviors are generally considered more severe than non-aggressive behaviors; however, all behaviors are given equal weight in the scoring of the measure (Cohen-Mansfield, 1991).

The CMAI was originally designed for use by professional care givers in institutional care settings (Cohen-Mansfield, 1991). But Alzheimer's dementia is associated with a significant burden on family members, who provide the majority of care, and other informal care partners (Leroi et al., 2007; Press and Buss, 2023). Our qualitative study therefore aimed to assess the accuracy and applicability of the CMAI according to the needs and perceptions of non-professional care partners. We sought to determine whether the CMAI is an appropriate instrument to assess changes in agitation behaviors in people with Alzheimer's dementia (AD) in the community setting, and to elicit what care partners consider to be a meaningful change in agitation behaviors. In addition, we aimed to understand the concordance between the frequency and severity of agitated behaviors.

## 2 Materials and methods

The research consisted of a qualitative, non-interventional, descriptive, cross-sectional study of non-professional care partners of patients with AD living in the United States. Researchers conducted semi-structured interviews with 30 non-professional care partners.

Care partners were invited to take part in the study if they were at least 21 years of age, cared for a person with clinically confirmed AD between 55 and 90 years old, spent at least 2 h per day on at least 4 days per week with the person with AD, were willing and able to participate in a 60–90 minute interview in English, and noticed any of the following behaviors frequently within the past 2 weeks in the person with AD: emotional distress; excessive movements (e.g., pacing, rocking); verbal aggression (e.g., yelling, using profanity); physical aggression (e.g., grabbing, shoving); and resulting negative impacts on their social and everyday functioning.

Non-professional care partners included family care partners and those who provide unpaid, informal care for patients with AD. UsAgainstAlzheimer (UsA2) recruited participants via their A-LIST^®^ panel of AD patients and care partners. A total of 912 panel members received an email invitation to participate in the study. Interested care partner participants completed a screening questionnaire to determine eligibility and provided informed consent. The study was centrally reviewed by Advarra IRB (IRB #Pro00049935). Interviews were conducted between May 5, 2021, and June 14, 2021, by three trained interviewers.

### 2.1 Conduct of interviews

Interviews were conducted virtually and were transcribed. They were conducted, one-on-one, following a semi-structured interview guide. Each participant was scheduled for a 60-min research session. Research sessions included (a) the completion of a background information form (demographics, health status, caregiving situation), (b) the CMAI via REDCap, an online survey management platform, followed by (c) an interview to discuss their experience with agitation behaviors and the relevance of the CMAI items (behaviors), main concepts, and domains as it relates to their caregiving experience.

The interviews asked open-ended questions about agitation, including how agitation presented and what they considered to be the most and least bothersome behaviors. Targeted questions were used to gather information about behaviors covered by the CMAI if they were not spontaneously discussed by participants. Participants were asked, for example, about the duration, frequency, and severity of both spontaneously elicited and prompted behaviors. Participants were asked to discuss what a meaningful improvement and/or meaningful decline in agitation would look like from the care partner's perspective. Participants were also asked to discuss what a meaningful improvement and/or meaningful decline in agitation (to them) would look like for the person with AD.

### 2.2 Coding and analysis

Interviews were recorded and transcripts were generated from the recordings by an independent transcription agency. Transcripts were reviewed by transcription agency senior staff for accuracy, then reviewed again by Modus Outcomes personnel before coding began. Interview transcripts were analyzed thematically (Bryman and Burgess, 2022) through detailed line-by-line inductive (Thomas, 2006; Bowling, 2009) and deductive coding (Crabtree and Miller, 1999) using ATLAS.ti software. To ensure consistency in coding, the first interview transcript was coded independently by two researchers (TL, AL), who then met to compare their results and align on methods going forward. Codes were organized based on existing CMAI factor structures, as well as the severity of behaviors, frequency of behaviors, and meaningful change. The coding was revised to reach coder agreement and following the identification of new concepts in the remaining transcripts. Codebook revisions were performed in consultation with a senior researcher (DO) as needed to reach coder agreement (e.g., related to specific wording or formatting). The codebook was further developed as new concepts were identified in the remaining transcripts.

## 3 Results

### 3.1 Care partner characteristics

We interviewed 30 care partners whose characteristics are described in Table 1. The majority were female (70%) and Caucasian (77%) with a mean age of 64 (±13) years. Just over half of the participants (53%) provided care for a spouse and 47% provided care for a parent or parent-in-law. Most care partners (77%) reported providing over 40 h of care per week.

**Table 1:** Care partner characteristics.

**Demographics**		**Educational and employment status**		**Caregiving**	
**Age (years)**		**Education level**, ***n*** **(%)**		**Relationship to PATIENT**, ***n*** **(%)**	
Mean (SD)	64 (13)	Highschool /GED	0 (0)	Partner/Spouse	16 (53)
**Sex**, ***n*** **(%)**		Some college	6 (20)	Parent (in Law)	14 (47)
Female	21 (70)	Associate degree	0 (0)	**Lives with patient**, ***n*** **(%)**	
Male	9 (30)	Bachelor's degree	7 (23)	Yes	26 (87)
**Race**, ***n*** **(%)**		Post-graduate	17 (57)	No	3 (10)
White	24 (77)	**Employment**, ***n*** **(%)**		Missing	1 (3)
Black	4 (13)	Part-time	5 (17)	**Types of Care**, ***n*** **(%)**	
Native American	1 (3)	Full-time	7 (23)	Companionship	29 (97)
Asian	1 (3)	Retired	13 (43)	Transportation	26 (87)
Multiracial	1 (3)	Homemaker	3 (10)	Homemaking	26 (87)
**Ethnicity**, ***n*** **(%)**		Not employed	2 (7)	Personal care assistance	26 (87)
Non-Hispanic	28 (93)			Healthcare assistance	26 (87)
Hispanic	1 (3)			Financial assistance	25 (83)
Missing	1 (3)			**Caregiving hours per week**, ***n*** **(%)**	
**Marital status**, ***n*** **(%)**				1-5h	1 (3)
Single	7 (23)			6-20h	2 (7)
Married	21 (70)			21-40h	4 (13)
Divorced/Separated	1 (3)			40+h	23 (77)
Missing	1 (3)				

Age missing for four care partners.

**Figure 1:**
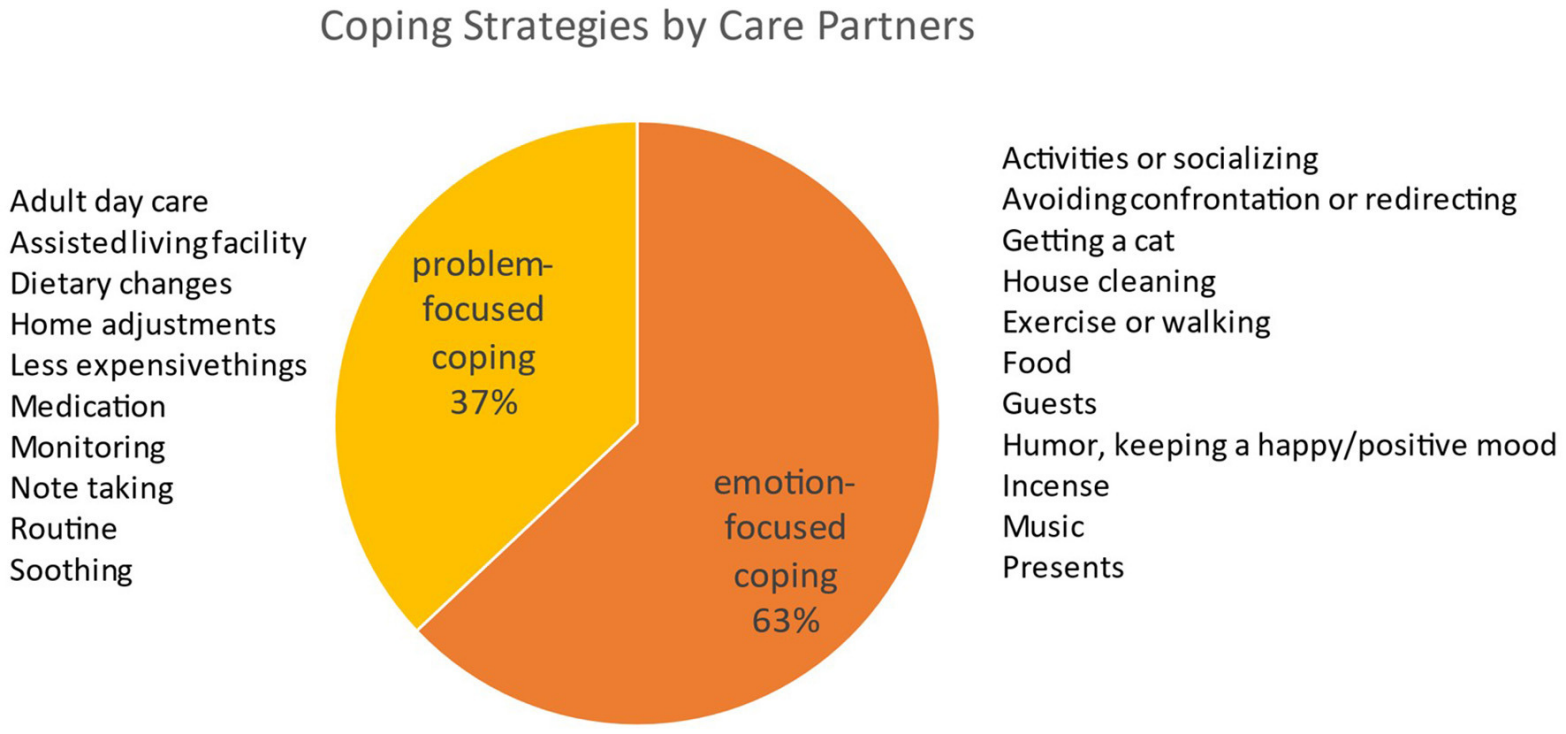
Coping strategies by care partners.

Care partners reported a variety of coping strategies to help them deal with the agitation behaviors, which were summarized under two broad categories: emotion-focused or problem-focused strategies (Figure 1). General coping strategies employed by caregivers included finding ways to relax or be relieved of their caregiving burden periodically. Caregivers also reported specific strategies they used to deal with the agitation behavior in specific situations; these included using humor to diffuse situations, refocusing, trying to implement changes to prevent recurrence of behaviors or mitigate behavior impacts.

### 3.2 Agitation behaviors

**Figure 2:**
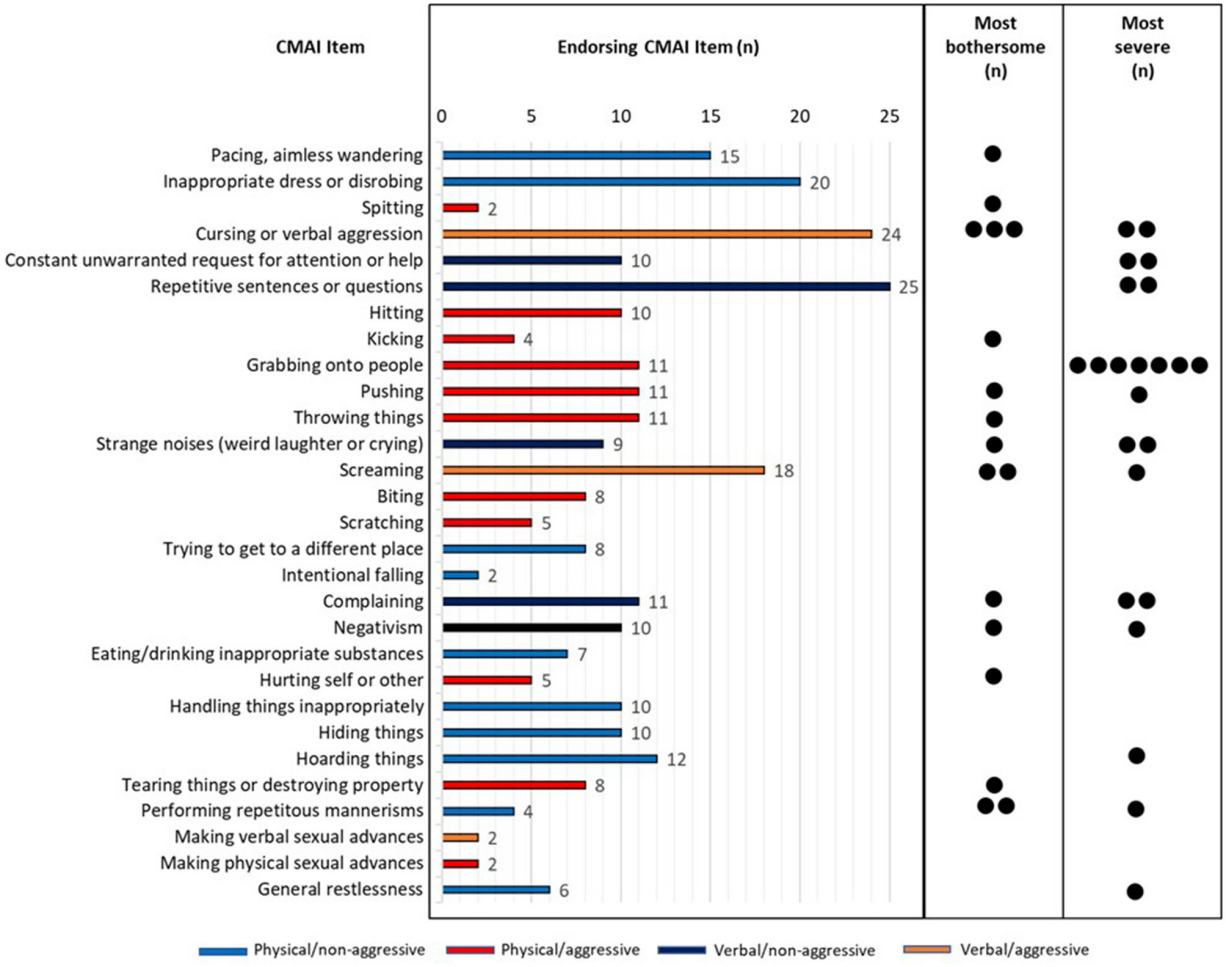
Frequency of CMAI items endorsed by care partners.

All behaviors (items) within the CMAI were endorsed by participants. Figure 2 provides a summary of the frequency of CMAI items that were endorsed by care partners, as well as those behaviors rated as the most bothersome or most severe. Care partner quotes to illustrate examples of behaviors and what constitutes improvement or worsening of agitated behavior are provided in Tables 2, 3.

**Table 2:** Participant quotes on experiences with behaviors.

Aggressive verbal	“For me [the screaming] bothers me a lot. It causes me a lot of stress … and then I'm afraid that it's going to escalate, and that puts fear in me that if it escalates and she tries to pick up something to hit me. So I try to make sure, I hate to say this, but I try to make sure there are no knives around or scissors or nothing she can pick up. Because I mean, I've read that that has happened to other's loved ones.” “But for her, it was like [she]'d never argued about anything, very mild-mannered … that was her thing – even-keeled. So just to raise your voice at somebody that you've never raised your voice with before and we are sisters and now you're 70 and I'm 76, what? For them, it's like something's wrong.” “So this yelling is something new for her. I don't recall ever being yelled at in our past years of marriage. Maybe once or twice, maybe at the kids, but that wasn't her mode of getting attention. It was very subtle. So this is new for me. It's a pretty loud scream.” “The agitation – the triggers for – she seems to be in a constant – sometimes it's low-level agitation. And sometimes it escalates – nothing like April 20th, though – the four hours of rocking and talking and shouting. And Mom speaks Spanish. She doesn't like to say she does. But when she gets agitated to the point where she starts to speak Spanish, I know that I've let her go too far. So that April 20th date, man she was full-fledged Spanish. There was no English in there. And she was talking to somebody on the couch. She was gesturing and talking. And after that, I was like no, we're not going to do this again. We are not doing this again. I have to mitigate circumstances, because this is not healthy – for her or for me. So I would say agitation and the stress associated with agitation, because she does get stressed afterwards. She falls asleep.”
Aggressive physical	“Well, it means a turning off toward whatever person might be trying to engage her, and she would wave her hands in the air or push strongly with one arm – you know, and deny any access. This happens very frequently when she is unengaged, say, before they would give her a medicine for her to take by a spoon that they would put at her mouth. If they had spoken to her without her engaging them face to face, she would be stiff armed and push. And this would happen even suddenly when I'm with her and I'm just holding her hand.” “Well, screaming and when she digs her fingernails into my hand because if she's doing it to me, she's also probably doing it to others. That could end some of her help. It's just not good. If she were hitting somebody, that would be bad. But she doesn't hit. Like I say, she uses her fingernails. So those two things. The yelling is just because it bothers my ears. But I'd rather have her yell than I would have her dig her fingernails in. But those are probably the two that are – I think, again, using her fingernails because it's basically an attack. It's a defensive attack. Simply saying no, simply not wanting to go – the other parts are frustrating and can cause more work for me, but I think a physical thing she does is probably the worst. As I said, it can create harm.” “The other thing also for your study, as that's what very important, is one of the things that's been a blessing is our cat and our cat has been absolutely miraculous. She is a 17-year-old cat and even starting from the period of time when my mother used to push me or throw things at me, my cat would come running into the room and stand between us or jump onto my lap to protect me. And as soon as my mother sees her, she melts and she's like oh, sweetest thing in the world. And when my mother is – also any time she's yelling, if my cat comes, that calms her down so very much. So she's totally been – our cat has been the one person in the family that she – that's been able to do things that we cannot do.” “She'll take her head, and she'll – I know what she's doing, so I kind of parry. I also took fencing years ago. I have pretty good reactions when someone swings at me. But she'll take her head, and she'll bang it against my head. And one time I really didn't realize that she was coming at me, and I didn't move fast enough. She clobbered me while I was getting her on a chairlift. It made me swing to the side. I had to hold on to the wall so I wouldn't fall over. Many, many times, I know where she's going with this. She almost starts to telegraph what she's doing.”
Non-aggressive verbal	“I would say the most severe with the behavior is the repeating questions and sentences. I think, over time, they have the most draining aspect from my energy.” “She'll say ‘They're terrible. These people are terrible,' and you're sitting right there and she's talking about under her breath and thinking you don't have any sense and you can't hear what she's saying. And it's very condescending, because I have someone in my house helping her and she's talking about the lady like a dog. So yeah, that bothers me. She has a lot of ways that I don't like, but the unappreciative ways is the worst one for me.” “I have whiteboards all over the house. And on the whiteboards, we talk about what's going to happen the next day, so I have the month, day and year. I have the day. And then I have a section that has appointments. So every morning and every night, she looks at that little whiteboard, both in her room and in the kitchen area, which is the central area, so that tells her what's going to happen that day. And if I – I found this out by accident – if I miss and don't change the date, she gets really upset. She's like, oh, you didn't change the date. Today's not Friday. Today's Saturday. How come you didn't change – and it's like, oh crap, it's a trigger. She gets agitated, because it's change.”
Non-aggressive physical	“Maybe moderately severe. I guess in terms of how often she [taps her fingers repetitively], it's very frequent, but I don't see her – it doesn't bring harm to her, it doesn't seem. So in that sense, I wouldn't be – I'm not as worried about it.” “I think it's she doesn't want to do what you're asking her to do. I'm not going to put a lot of why she's doing it, but I think, literally, she's just going limp. I think it's just – and sometimes she'll say I'm falling. But other times, I think she just simply – I don't know why. But all she's actually doing is – and I know she's doing this on purpose because she's quite capable. I've seen her stand in a place where I can't get her to move from. She has stood there for a half-hour. I mean, she had literally taken a stand. She's holding on to something, but she's on her feet for a very long period of time. So these times when she's just gone to the floor, I think it's a case of just – I think she just figures this is one way that she doesn't have to go somewhere. I think she does it just simply, so she's not forced to walk where we're trying to get her to walk, which is where she really wants to go anyway. I think it's intentional. I don't think the legs collapse because she's tired. I think it's a case of simply going limp. I really think it's intentional. ……… The only other time I saw it happening is when she was standing for an awful long time. And then she said, I'm getting tired. Then I could see the legs starting to waver, and I figured, let's just get her to sit wherever and deal with her that way.” “I would say it's mild because it doesn't bother me. I don't think I need to do anything about it. So I would say it's mild. I just make sure it's something that she can hold onto, hold in her hands.”

**Table 3:** Most bothersome behaviors and improvement vs. worsening.

**Most bothersome**	**Improvement vs. Worsening**
The constant aggression –waving her fist, spitting, kicking. Four years ago, she broke two of my toes. I was taking her to the doctor. And she'd given me a hard time getting in the car. And I'm like, Mom, you'll be out of the weather. Just get in the car. And she slammed her foot down. But for her, it was like I'd never argued about anything, very mild-mannered, very, very –like that was her thing –even-keeled. So just to raise your voice at somebody that you've never raised your voice with before and we are sisters and now you're 70 and I'm 76, what? (laughter) For them, it's like something's wrong. I'm going to say not at all [severe], because if that's the worst thing he does is take off a paper towel, fold it up and put it on his bedroom dresser, so what if I have to buy more paper towels? That's the least of my irritants or probably his. Maybe moderately severe. I guess in terms of how often she [taps her fingers repetitively], it's very frequent, but I don't see her –it doesn't bring harm to her, it doesn't seem. So in that sense, I wouldn't be –I'm not as worried about it. She'll say they're terrible. These people are terrible and you're sitting right there and she's talking about under her breath and thinking you don't have any sense and you can't hear what she's saying. And it's very condescending, because I have someone in my house helping her and she talking about the lady like a dog. So yeah, that bothers me. She has a lot of ways that I don't like, but the unappreciative ways is the worst one for me.	So I guess –well, an improvement would be, even if he reacts verbally, that he doesn't automatically grab and tense and clutch when he's surprised. An improvement would obviously mean that he just didn't do it, and a decline would probably be what I said –being a twisted old man that took off his clothes and tried to give the little old ladies a thrill. I guess that's it. Yeah, better is not doing it, and worse is doing it with intention. That he wouldn't do it. (laughter) He'd just roll with life. It'd be like, “eh”… in my life, what I call SWs, “so whats”? It would be an SW. Eh, OK. Life goes on. Not being able to calm her down at all. Having that anxiety constantly, and not being able to soothe her in any way. That would be –I don't know how I would handle that to be honest. [And if that yelling were to get worse,... ] I would think he'd be getting angrier more often and it would be more intense and he might be physical. My friends keep warning me, he's going to get physical.

#### 3.2.1 Verbal/aggressive behaviors

Care partners spontaneously endorsed all three items related to verbal/aggressive behaviors. Overall, care partners reported that verbal/aggressive behaviors had a high emotional impact on them, especially when seen as out of character for the patient prior to their diagnosis.

Cursing was the most frequently endorsed verbal/aggressive behavior and was perceived as severe due to being offensive and out of the fear that it would insult others, especially paid care partners. Screaming was reported as both a frequent (i.e., occurring at least once per week) and severe behavior. Screaming was also reported as bothersome to care partners, as some feared it would eventually escalate to physical aggression. Care partners did not describe verbal sexual advances as frequent or severe.

#### 3.2.2 Verbal/non-aggressive behaviors

All CMAI items pertaining to verbal/non-aggressive behaviors were spontaneously reported by at least one care partner. Repetitive sentences/questions were endorsed as the most frequent verbal/non-aggressive behavior and considered the most severe by two care partners. Constant unwarranted requests for attention/help, strange noises (e.g., weird laughter or crying), complaining, and negativism were reported by a similar number of care partners and were considered most bothersome and most severe by several care partners. Strange noises were described as severe but did not happen frequently, while constant unwarranted requests for attention/help and repetitive sentences/questions occurred frequently but were generally not seen as severe. Care partners also felt that all five behaviors varied in severity and frequency. The severity of these behaviors was related to the frequency and the duration of the behavior, patient responsiveness to intervention, emotional impact on both the care partner and patient, and consistency with the patient's personality.

#### 3.2.3 Physical/aggressive behaviors

Each of the CMAI items pertaining to physical/aggressive behaviors was spontaneously endorsed by care partners. These behaviors were not described as occurring frequently but were perceived as severe when they did occur. All behaviors but two (scratching and making physical sexual advances) were described as bothersome or severe, and this perception of severity was based on the potential for physical harm and a need for a higher level of care/support to handle.

Care partners also felt that physical/aggressive behaviors were often triggered by an unwanted stimulus. The specific stimuli cited by caregivers included a generalized loss of independence or loss of control over situations (e.g., being told what to do, receiving criticism, not being able to handle delayed gratification, not feeling useful); denial or lack of acceptance of the Alzheimer's diagnosis; overreaction to emotional and/or physical stimuli, and deviation from routine. The emotional and/or physical stimuli could be real, imagined, in real-life, or on television.

#### 3.2.4 Physical/non-aggressive behaviors

The only behavior item on the CMAI that was not spontaneously reported was intentional falling (*n* = 2, when probed). Care partners reported frequent falls, but they were not usually reported as intentional. Instead, falling was due to a loss of balance or a loss of strength due to other agitation behaviors (e.g., stubbornly standing stock-still for extended periods leads to them falling over or collapsing). Caregivers also reported falls due to fainting spells (syncope). In the few cases where intentional falling was reported, the behavior was described as a form of opposition or refusal to follow a request by the caregiver.

All other physical/non-aggressive behaviors were endorsed spontaneously by at least one care partner. Physical/non-aggressive behaviors varied in frequency, but many were described as occurring at least once per week. These behaviors also varied in severity, and their severity was characterized by their duration, the level of attention required from the care partner, and their impact on daily life.

### 3.3 Meaningful change in agitation behaviors

**Figure 3:**
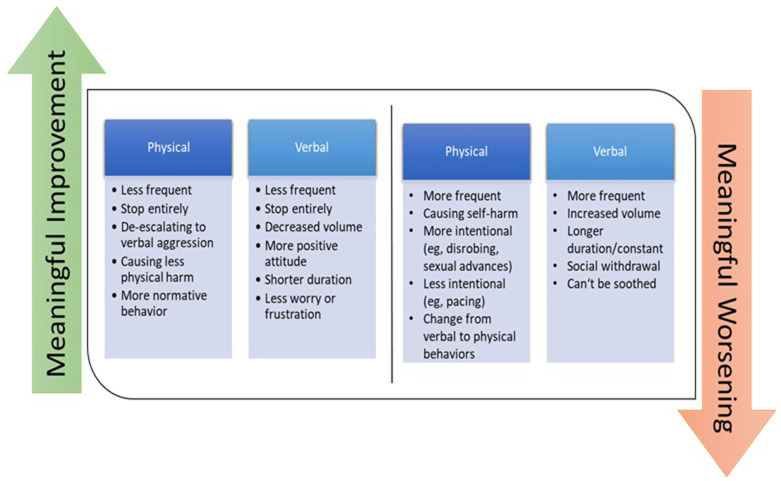
Meaningful improvement or worsening of physical and verbal agitation behaviors according to care partners.

Figure 3 shows how care partners related physical and verbal behaviors (specific behaviors or overall) to a meaningful improvement or worsening of agitation. All care partners tied a meaningful change in a CMAI agitation behavior to a change in the frequency and/or the intensity of the behavior. Additionally, care partners felt that the amount of harm caused (e.g., physical aggression, intention to harm) was related to the perception of the behavior's severity. Physical behaviors were generally considered to be more severe than verbal behaviors. Changing from verbally aggressive to physically aggressive behaviors was considered an escalation and meaningful worsening. A shift from mostly physical aggressive behaviors to verbally aggressive was perceived as an improvement. Changes in frequency, volume, and duration were additional dimensions of the perceived improvement or worsening for verbal behaviors reported by care partners. Overall, care partners viewed any reduction as a meaningful improvement, whereas an increase was viewed as a meaningful worsening.

## 4 Discussion

The objective of this study was to contribute to the evaluation of the CMAI as a fit-for-purpose instrument for assessing changes in agitation in people with AD and to evaluate meaningful changes from the care partner perspective. More specifically, the study aimed to evaluate the CMAI with respect to the needs and perspectives of family members and other informal and non-professional care partners; the study was designed to assess the CMAI in a community setting, rather than the institutional context for which it was originally designed and intended.

More specifically, the study aimed to evaluate the CMAI with respect to the needs and perspectives of family members and other informal and non-professional care partners; the study was designed to assess the CMAI in a community setting, rather than the institutional context for which it was originally designed and intended. Our confirmation of the CMAI's fit for purpose in this sample and setting is important for several reasons. Firstly, unpaid caregivers tend to have fewer supporters and thus bear a disproportionate burden of worsening agitation—and may especially benefit from its improvement. Secondly, agitation triggers may differ between institutional and community settings, but the relevance of CMAI agitation behaviors across settings is a useful finding. Third, as more people with dementia reside at home and in the community, relevant measures of clinical progression and treatment benefit in this setting are needed.

Although patients may not display every behavior accounted for in the CMAI, care partners endorsed all items in the instrument, finding them easy to understand and reflective of their caregiving experience with agitation in AD. Items varied in perceived severity, which reflected the manner in which care partners discerned between meaningful improvement and meaningful worsening. Care partners overwhelmingly reported changes in the frequency of the behavior to be meaningful, with increasing frequency considered a worsening and a decrease in frequency considered an improvement.

While any reduction in agitation behaviors was considered preferable, care partners emphasized that there are differences between physical and verbal behaviors, especially when it comes to the perception of their severity. Overall, physical behaviors were perceived as more severe than verbal behaviors; caregivers also noted that verbal behaviors can escalate into physical behaviors: Care partners reported that the severity of agitation behaviors was multi-factorial and related to the frequency and duration of the behavior. Regarding the concept of symptom severity, care partners emphasized the following: changes in personality (e.g., from at a state of “normal” passive-aggressiveness to one of sustained aggressive verbal outbursts); changes in volume [an increase in aggression might encompass both an increase in degree of verbalization (shouting/yelling/screaming) or a decrease in volume (whispering, intentionally speaking not loud enough)]; changes in tone/voice character (e.g., from calm to aggressive/harsh); changes in speed (often faster more agitated or escalating); changes in pronunciation or enunciation; language switching (e.g., switching from habitually speaking English to Spanish). The concepts of agitation behavior severity and frequency are interrelated for care partners, and the more severe and bothersome the behaviors, the more disruptive they were to care partners with the highest severity attributed to behaviors that were harmful.

However, we urge caution in interpreting the severity of a patient's agitation from reports of the frequency of behaviors. The CMAI was initially created to assess behavior frequency, and its developers acknowledge that some behaviors are inherently more severe than others (Cohen-Mansfield, 1991). This is supported by our findings that: (a) care partners consider physical behaviors as more severe than verbal behaviors, (b) physical behaviors can meaningfully decline to verbal behaviors, and, conversely, that (c) verbal behaviors can meaningfully worsen to physical behaviors. A large amount of heterogeneity of observed behaviors on the CMAI is therefore to be expected since no patient will exhibit all behaviors at any given time and patients will move along a spectrum of behaviors as their agitation improves or worsens. These results suggest that a sum of all CMAI items to form a total score may not be a complete reflection of agitation severity, improvement, or worsening. Rather, as suggested in the CMAI manual, the means of aggregating CMAI items should be conceptually driven to account for underlying latent constructs that are relevant to the population being studied (Cohen-Mansfield, 1991).

### 4.1 Limitations

While this study did produce robust data concerning the appropriateness the CMAI as a fit-for-purpose instrument for assessing changes in agitation in people with AD in the context of non-institutional settings and from the perspective of non-professional care partners, there were some limitations. The study relied on a small sample size of care partners, which was racially homogenous and lacked economic and educational diversity. Additionally, the heterogeneity of agitation behavior manifestations may have been magnified by the small sample size. Future studies should recruit a larger, more diverse sample to investigate the nuances of care partner experiences and to ensure that the CMAI accurately assesses AD agitation in a more diverse population of community caregivers.

## 5 Conclusion

Overall, our research confirms the CMAI's content validity and appropriateness for assessing changes in agitation behaviors for people with AD. The CMAI captures concepts and behaviors which are relevant to care partners and use of the instrument can provide insights into agitation severity and meaningful changes in agitation behaviors. Considering the substantial care partner burden resulting from agitation behaviors, use of a tool like the CMAI by care partners and clinicians alike could help identify problems earlier in the hope of mitigating escalation to more severe outcomes for both patients and their care partners.

## Data availability statement

The datasets presented in this article are not readily available because participant consent only covered use for the specific research purpose. Requests to access the datasets should be directed to https://clinical-trials.otsuka.com.

## Ethics statement

The studies involving humans were approved by the Advarra Institutional Review Board. The studies were conducted in accordance with the local legislation and institutional requirements. The participants provided their written informed consent to participate in this study.

## Author contributions

DO: Conceptualization, Formal analysis, Funding acquisition, Investigation, Methodology, Project administration, Resources, Supervision, Validation, Visualization, Writing – original draft, Writing – review & editing, Data curation. AP: Investigation, Methodology, Project administration, Supervision, Writing – review & editing. CH: Conceptualization, Funding acquisition, Methodology, Writing – review & editing. TL: Data curation, Formal analysis, Investigation, Methodology, Project administration, Writing – review & editing. AL: Data curation, Formal analysis, Investigation, Methodology, Project administration, Writing – review & editing. TF: Conceptualization, Methodology, Resources, Supervision, Validation, Writing – review & editing. GB: Conceptualization, Resources, Supervision, Writing – review & editing. MC: Conceptualization, Supervision, Writing – review & editing.
